# Evo-physio: on stress responses and the earliest land plants

**DOI:** 10.1093/jxb/eraa007

**Published:** 2020-01-10

**Authors:** Janine M R Fürst-Jansen, Sophie de Vries, Jan de Vries

**Affiliations:** 1 University of Göttingen, Institute for Microbiology and Genetics, Department of Applied Bioinformatics, Göttingen, Germany; 2 Population Genetics, Heinrich-Heine University Düsseldorf, Düsseldorf, Germany; 3 University of Göttingen, Göttingen Center for Molecular Biosciences (GZMB), Göttingen, Germany; 4 University of Osnabrück, Germany

**Keywords:** Charophytes, earliest land plants, exaptations, plant evolution, plant terrestrialization, streptophyte algae, stress physiology, terrestrial algae

## Abstract

Embryophytes (land plants) can be found in almost any habitat on the Earth’s surface. All of this ecologically diverse embryophytic flora arose from algae through a singular evolutionary event. Traits that were, by their nature, indispensable for the singular conquest of land by plants were those that are key for overcoming terrestrial stressors. Not surprisingly, the biology of land plant cells is shaped by a core signaling network that connects environmental cues, such as stressors, to the appropriate responses—which, thus, modulate growth and physiology. When did this network emerge? Was it already present when plant terrestrialization was in its infancy? A comparative approach between land plants and their algal relatives, the streptophyte algae, allows us to tackle such questions and resolve parts of the biology of the earliest land plants. Exploring the biology of the earliest land plants might shed light on exactly how they overcame the challenges of terrestrialization. Here, we outline the approaches and rationale underlying comparative analyses towards inferring the genetic toolkit for the stress response that aided the earliest land plants in their conquest of land.

## Teaser

During plant terrestrialization, the earliest land plants had to adequately respond to environmental stressors; combining insights from streptophyte algae and embryophytes allows inferences of how they succeeded in doing so.

## Introduction

### Green evolution: from the origin of photosynthetic eukaryotes to the earliest land plants

**Fig. 1.**
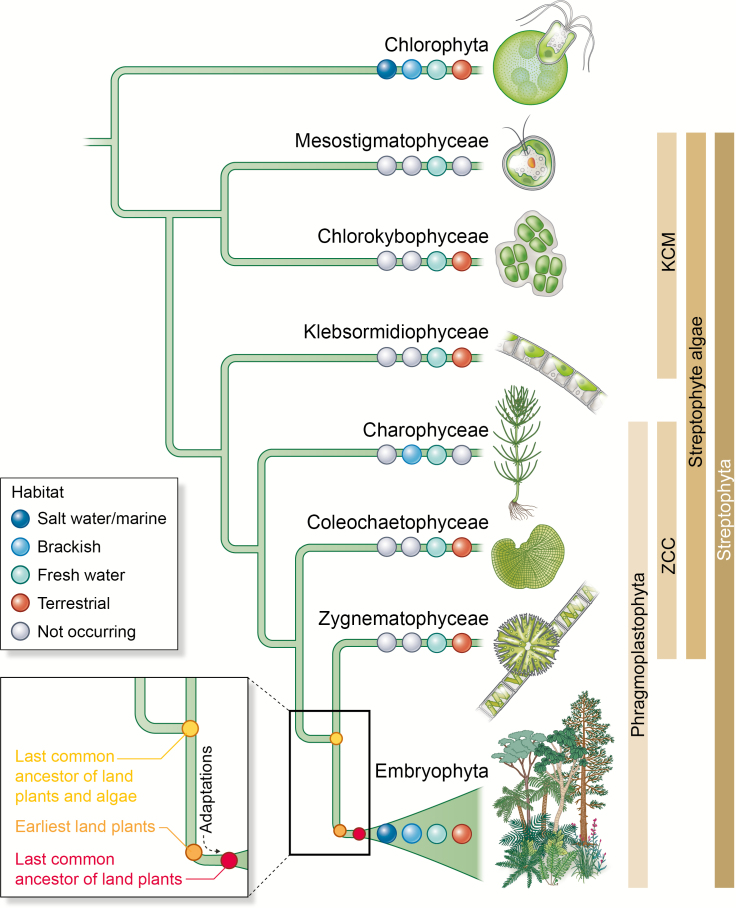
Terrestrial organisms are found across the green lineage. A cladogram shows the deep split of the green lineage into the clades Chlorophyta and Streptophyta. The Streptophyta are composed of the paraphylum streptophyte algae and the monophyletic Embryophyta (land plants). Streptophyte algae can be broken up into the paraphyla KCM (for Klebsormidiophyceae, Chlorokybophyceae, and Mesostigmatophyceae) and ZCC (for Zygnematophyceae, Coleochaetophyceae, and Charophyceae; de Vries *et al.*, 2016). ZCC streptophyte algae and land plants form the monophyletic clade Phragmoplastophyta. Taken in their entirety, Chlorophyta occur in habitats ranging from marine saltwater, to freshwater, to terrestrial (row of dots). Streptophyte algae mainly occur in freshwater and terrestrial environments; some Charophyceae live in a brackish habitat. While the Embryophyta are mainly terrestrial, some have secondarily moved back to a freshwater habitat; some have even conquered a new habitat: saltwater (e.g. sea grasses). Inset: the Zygnematophyceae are the closest algal relatives of land plants and they hence share with the clade of Embryophyta the last common ancestor of land plants and algae (yellow dot); along the trajectory from that last common ancestor of land plants and algae (yellow dot) to the last common ancestor of land plants (red dot) are the earliest land plants to be found (orange dot). Inferring the biology of the earliest land plants requires a subtraction of the traits (‘adaptations’) that were gained on land, that is en route to the last common ancestor of land plants (from the orange to the red dot; see also Fig. 2).

Photosynthetic eukaryotes probably first emerged >1.5 billion years ago (Butterfield, 2000; Eme *et al.*, 2014; Bengtson *et al.*, 2017). Underlying the origin of photosynthetic eukaryotes was the endosymbiotic uptake of a free-living cyanobacterium by a heterotrophic protist—an event that gave rise to the Archaeplastida (reviewed by Archibald, 2015; Martin *et al.*, 2015; de Vries and Gould, 2018). There are three types of Archaeplastida: the red algae (rhodophytes), the glaucophytes, and the green lineage (Keeling, 2013; Archibald, 2015; Jackson *et al.*, 2015). The green organisms make up the Chloroplastida (Fig. 1)—a name that should be given preference over the previous label for that clade, Viridiplantae (Adl et al., 2005, 2019). Within the Chloroplastida, we find both green algae and the land plants (reviewed by Leliaert *et al.*, 2012; de Vries *et al.*, 2016).

The green lineage separated roughly a billion years ago into the chlorophytes and the streptophytes (Zimmer *et al.*, 2007; Parfrey *et al.*, 2011; Morris *et al.*, 2018). While the chlorophytes are generally perceived as the clade comprising famous green algae (such as *Volvox*, *Ulva*, and *Chlamydomonas*), the streptophytes are best known as the clade containing the land plants. However, there is more to the lineage of streptophytes. In the phylogeny of streptophytes sits—next to the land plants—the paraphylum of streptophyte algae (Fig. 1). It is this grade that one must turn to in order to understand the origin of land plants.

**Fig. 2.**
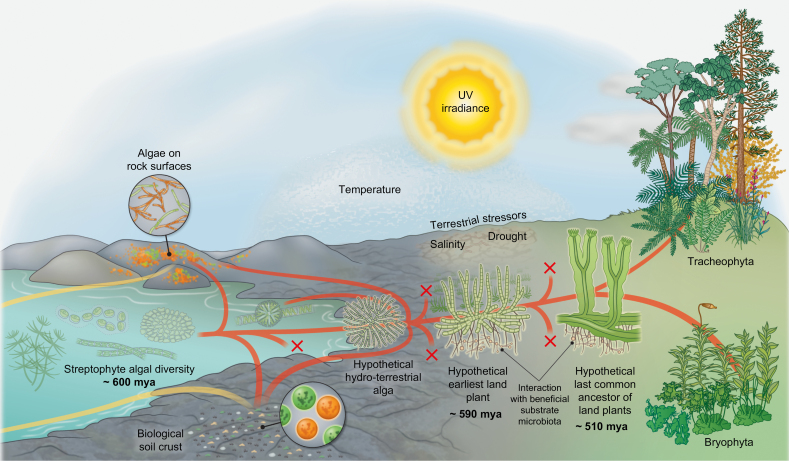
The earliest land plants: an evolutionary scenario for the conquest of land by streptophytes. Streptophyte algae are the only photosynthetic eukaryotes from which the macroscopic land flora evolved (red lines). That said, throughout the course of evolution, algae from various other lineages have colonized land (yellow lines)—but also streptophyte algae have continuously and independently made the wet to dry transition (convergence of red and yellow). Throughout history, numerous lineages have become extinct (‘x’ labels). Terrestrial algae of various taxonomic affiliations dwell on rock surfaces and form biological soil crusts. From the diversity of the paraphyletic streptophyte algae, however, did an organism whose descendants eventually conquered land on a global scale emerge: a likely branched filamentous—or even parenchymatous—organism that formed rhizoidal structures and experienced desiccation from time to time. From this ‘hypothetical hydro-terrestrial alga’, the lineages of Zygnematophyceae and embryophytes (land plants) arose. In its infancy, the trajectory leading to the embryophytes was represented by the—now extinct (see also Delaux *et al.*, 2019)—earliest land plants. The earliest land plants probably interacted with beneficial substrate microbiota that aided them in obtaining nutrients from their substrate. Furthermore, the earliest land plants had to successfully overcome a barrage of terrestrial stressors (including UV and photosynthetically active irradiance, drought, drastic temperature shifts, etc.). They succeeded because they had the right set of traits—a mix of adaptations that were selected for in their hydro-terrestrial algal ancestors, exaptations, and the potential for co-option of a fortuitous set of genes and pathways. During the course of evolution, some members of the populations of the earliest land plants gained traits that are adaptive in terrestrial environments (such as some form of water conductance, stomata-like structures, embryos, etc.); eventually, the ‘hypothetical last common ancestor of land plants’ emerged. From this ancestor, the extant bryophytes and tracheophytes evolved. While the exact trait repertoire of the hypothetical last common ancestor of land plants is uncertain, it will certainly have entailed properties of vascular and non-vascular plants. What is also certain is that the last common ancestor of land plants had traits of algal ancestry. (All dating is roughly based on Morris et al. [2018])

All land plants evolved from a single streptophyte algal progenitor (reviewed in de Vries and Archibald, 2018). The streptophyte algae are a group of mainly freshwater and terrestrial algae—with a few representatives living in brackish environments (Lewis and McCourt, 2004; Becker and Marin, 2009; Fig. 1). That the streptophyte algal ancestors of land plants lived in freshwater—as opposed to marine—environments is considered a major factor leading to terrestrialization: starting from a freshwater environment such as a pond, there was smooth passage along the hydrological gradient towards land (see discussions in Becker and Marin, 2009; Delwiche and Cooper, 2015; de Vries and Archibald, 2018). It is this stepwise conquest of land along the hydrological gradient where the earliest land plants—or the first common ancestors of land plants—are evolutionarily and ecologically situated (Figs 1, 2). This ecological setting, however, does not constitute a reason for the singular global conquest of land by streptophytes. Indeed, photosynthetic eukaryotes might have had a freshwater origin (Delwiche and Cooper, 2015; de Vries and Archibald, 2017; Lewis, 2017; Ponce-Toledo *et al.*, 2017; Sánchez-Baracaldo *et al.*, 2017). Streptophytes are also not the only photosynthetic eukaryotes that dwell in freshwater environments and on land. Various other algae, including chlorophytes (for an overview, see Holzinger and Karsten, 2013), diatoms (for an overview, see Souffreau *et al.*, 2013), red algae (e.g. *Porphyridium*, see John, 1942), and many more, are terrestrial, too (Hoffmann, 1989). Hence, the question is not only what allowed for the origin of land plants but also what allowed for their unique success—a success that resulted in the global conquest of land. In this review, we will explore the complexities underlying these questions and make a case for dissecting one—which is by far not the only—key aspect of the biology of the earliest land plants: adequately responding to terrestrial stressors.

### Synapomorphies and the global success of land plants

Embryophytes (land plants) are defined by a series of traits. For example, land plants undergo a cycle where they alternate between a diploid sporophyte and a haploid gametophyte. An alternation of generations that involve two multicellular generations of different ploidy is one of the features of land plants that sets them apart from their algal ancestors (for more on this topic, see Bowman *et al.*, 2016; Horst *et al.*, 2016; Rensing, 2018). Among such embryophytic traits, we also might find those features that allowed for the success of the monophylum of land plants.

As the name implies, having embryos and embryogenesis is a signature feature of embryophytes. Broadly speaking, the embryo is a parentally supported complex structure with different tissue types. The exact organization and structure of embryos varies across the diversity of land plants concomitant with the dominance of sporophyte and gametophyte (for an overview of the underlying process, see Rensing, 2016). Support both through the parent organism and the structural framework that the embryo defines is thought to have been adaptive for living on land: the pre-defined structure of the embryo leads to an upright body plan with specialized tissues, both of which can foster nutrient uptake and nuanced responses to environmental cues via specialized cells (Rensing, 2016). When pondering the evolution of plant embryos, the seed of spermatophytes comes to mind. The seed is a structure that is highly resistant to terrestrial stressors (principally desiccation—see stimulating discussions in Oliver *et al.*, 2000). Seeds obtain their stress resistance through molecular mechanisms such as the accumulation of LATE EMBRYOGENESIS ABUNDANT (LEA; e.g. Dure *et al.*, 1981; Xu *et al.*, 1996) proteins and seed dormancy regulation via abscisic acid (ABA; reviewed in Holdsworth *et al.*, 2008); more on this below. While the seed clearly is a derived structure of spermatophytes, the molecular framework that underpins their stress resilience is probably conserved across the breadth of land plant diversity (Cuming *et al.*, 2007; Eklund *et al.*, 2018). This framework was hence likely to have been present in the last common ancestor of land plants—and potentially even before.

Land plants have evolved a number of complex structures that are adaptive in a terrestrial habitat (Harrison, 2017). Among these structures are stomata. There is much debate about the exact trajectory of stomata evolution; for an overview, see Chater *et al.* (2017). Yet, it is considered probable that some sort of stomata were a feature of the last common ancestor of land plants (e.g. Duckett and Pressel, 2018). The ancestral function of stomata is, however, ambiguous and much debated (see, for example, Duckett and Pressel, 2018; Pressel *et al.*, 2018). A similar case applies to water-conducting tissues. Land plants need to allocate water from their substrate, which is facilitated by rooting structures that range from rhizoids in non-vascular plants to the ‘true roots’ of vascular plants (reviewed by Jones and Dolan, 2012; Kenrick and Strullu-Derrien, 2014; Hetherington and Dolan, 2017). Root-mediated water conduction from the substrate through the entire plant is a textbook process of vascular plants that is clearly adaptive in aeroterrestrial environments. Some mosses and liverworts have water-conducting tissues such as hydroids—yet these probably evolved multiple times independently (Ligrone *et al.*, 2000) and most do not have water-conducting tissues. Surprisingly, Xu *et al.* (2014) highlighted that the same group of transcription factors (TFs), the NACs, that regulate xylem differentiation in the vascular plant *Arabidopsis thaliana* also regulate hydroid differentiation in the moss *Physcomitrella patens*. This has potential implications for vascular or non-vascular water-conducting cells in the last common ancestor of land plants (see Fig. 2). The findings of Xu *et al.* (2014) thus underscore the genetic capacity for the earliest land plants to have gained a complex system for water conduction.

This list could be continued but there is a stumbling block to most of the above-named traits: they define land plants as we know them today. These traits were likely to have been present in the last common ancestor of all land plants. Yet, it is difficult to put the gain of these traits into the right order that might enable us to reconstruct a scenario for the origin of land plants. Here the closest streptophyte algal relatives of land plants can help. Among streptophyte algae, we find (i) traits that were once classified as land plant specific and (ii) genes that are required for realizing such traits—even if they are not fully realized in the algae or used in an entirely different manner as in land plants.

### Inferring trait evolution towards understanding the singularity of plant terrestrialization

The last common ancestor of land plants was an embryophyte. As elaborated in the previous paragraph, this common ancestor must have had an array of the synapomorphic traits that define embryophytes. These traits give a post-hoc perspective on the singularity of the origin of the embryophytic clade: The last common ancestor was probably already established on land. However, when did the decisive traits evolve if we consider the earliest land plants and, thus, the organisms that conquered land (see Fig. 2)? To pinpoint those features that might have allowed for the conquest of land, we have to look at what happened before the last common ancestor of land plants lived—we have to resolve features of the biology of the earliest land plants (Figs 1, 2). To do so, we need to add an informative set of streptophyte algae to the picture.

In the past few years, garnering phylogenetic (e.g. Wickett *et al.*, 2014; Puttick *et al.*, 2018; Leebens-Mack *et al.*, 2019) and sequence data (e.g. Hori *et al.*, 2014; Delaux *et al.*, 2015; Ju *et al.*, 2015; de Vries *et al.*, 2018a; Nishiyama *et al.*, 2018) for streptophytes has gained momentum, resulting in a changing picture of early land plant evolution. In light of these new data, the notion that the embryophyte lineage might be split into two monophyletic groups (Puttick *et al.*, 2018) raises the question of what the properties of the common ancestor of embryophytes might be; its repertoire of traits could have entailed features of both bryophytes (mosses, liverworts, and hornworts) and tracheophytes (vascular plants). Here, a streptophyte algal perspective will help, too. Disentangling the transition from the earliest common ancestor to the last common ancestor of land plants (see also Fig. 2) will not only illuminate the properties present and relevant during the earliest steps of plants on land, but also those at the base of the land plants.

In the previous section, we have listed a number of adaptations of embryophytes to living on land. If we projected their origin onto the trajectory from streptophyte algal ancestor to extant embryophytes, we would find that the streptophyte algal progenitor probably possessed a few of these key traits—at least in a rudimentary fashion. Palpably, these include rhizoids and multicellular growth.

Rhizoids or similar structures can be found in all phragmoplastophytic streptophyte algae. The most obvious cases of these are the multicellular rhizoids of Charophyceae, whose statoliths are even involved in the modulation of gravitropism (e.g. Leitz *et al.*, 1995). The Coleochaetophyceae do not form rhizoids, but have special hairs that rest in a sheath, whose possible homology with rhizoids was discussed by Graham *et al.* (2012). Among the Zygnematophyceae, which are phylogenetically most closely related to land plants, rhizoid formation for providing anchorage to a substrate has been meticulously described in *Spirogyra* (Yoshida *et al.*, 2003; Ikegaya *et al.*, 2008; Yoshida and Shimmen, 2009). Rhizoid formation is, hence, a probable feature of the earliest land plants (Fig. 2).

Multicellularity in phragmoplastophytic streptophyte algae can be supported by a simplex meristem/apical cell structure. Charophyceae have an apical cell at the tip of the shoot-like structure that confers its erect growth (Pickett-Heaps, 1967). In *Coleochaete*, varying growth morphologies occur that range from branched filaments to discoidal parenchymatous growth (for an overview, see Delwiche *et al.*, 2002); morphogenesis of the latter is underpinned by meristems (Dupuy *et al.*, 2010). During the course of evolution, the zygnematophyceaen body plan probably experienced some reduction—as, for example, seen in the desmids that reverted to unicellularity. However, as already indicated previously, some filamentous Zygnematophyceae have a few morphological surprises up their sleeves, including the formation of rhizoidal holdfasts (e.g. Ikegaya *et al.*, 2008) and branching (e.g. Stancheva *et al.*, 2014). Altogether, this suggests that the earliest land plants probably had a body plan that entailed at least branching filaments—if not parenchymatous growth—likely to resemble to some degree the body plans found among the diversity of the genus *Coleochaete* (Fig. 2; Delwiche and Cooper, 2015). A multicellular body plan facilitates the differentiation of cells that can specialize in the responses to environmental stressors, for example the uppermost layer of a hypothetical body plan being particularly rich in compounds that act as sunscreens. Thus, multicellularity might have provided selective advantages for the earliest land plants when facing stress on *terra firma*.

### Genetic potential and the evolution of decisive traits

The embryophytic transcriptionally active protein (TAP) repertoire has often been proposed to explain the complex developmental phenotypes and high plasticity in environmental responses of land plants (Lang *et al.*, 2010; Mähönen *et al.*, 2014; Harrison, 2017; Scheres and van der Putten, 2017; Wilhelmsson *et al.*, 2017). Recent studies (Catarino *et al.*, 2016; Wilhelmsson *et al.*, 2017), however, showed that >80% of the TAP family repertoire of land plants is present in streptophyte algal genomes and transcriptomes. It may therefore be assumed that an interconnected network of TAPs and their downstream targets already fine-tuned the biology of the earliest land plants. Importantly, the TAP repertoire does more than the actualization of specific traits: TAPs create a fertile ground for genetic—and hence evolutionary—innovation through a few minor modifications such as spatial and temporal modification of the expression of a whole cascade. Thus, small changes in TF number, expression, or binding capacity can have profound effects on biological phenotypes. For example, the LOTUS JAPONICUS ROOTHAIRLESS LIKE (LRL) family in *A. thaliana* is separated into two antagonistically acting classes that have strong tissue-specific expression (Breuninger *et al.*, 2016). The antagonistic network in Arabidopsis gave rise to a control mechanism of a variety of morphological developments, while in *Marchantia polymorpha* one single copy gene regulates the development of rhizoids and thalli (Breuninger *et al.*, 2016). A directly stress-related example for the land plant-like TAP repertoire of streptophyte algae might be the GRAS TFs. GRAS TFs regulate both development and stress response of land plants (Hirsch and Oldroyd, 2009; B. Zhang *et al.*, 2018). In the recently reported genome sequence of the two Zygnematophyceae *Spirogloea muscicola* and *Mesotaenium endlicherianum* (Cheng *et al.*, 2019), the authors found that already the ancestor of Zygnematophyceae and land plants shared an expanded repertoire of genes coding for GRAS TFs. Some of these GRAS TFs are thus strong candidates for regulating conserved stress responses. Altogether, TAPs offer a plausible explanation for the rapid diversification in form and function (including stress-related) that we see across the diversity of land plants; the potential for this TAP-based radiation of form and function was already present in the algal relatives of land plants.

A tangible example of the fertile ground that the genetic material of streptophyte algae offers is the genetic toolkit to establish an interaction with symbiotic fungi (Delaux *et al.*, 2015). Most (>70%) of the extant diversity of embryophytes engage in symbiosis with arbuscular mycorrhiza that aid in obtaining nutrients from the substrate (Delaux *et al.*, 2014; Field and Pressel, 2018). Land plants use a core signaling toolkit to establish such symbioses (Parniske, 2008). Symbioses with beneficial substrate microbiota are thought to have aided the earliest land plants in gaining a foothold in the terrestrial habitat (Delaux *et al.*, 2013; Field *et al.*, 2015), and orthologous genes that are involved in upstream signaling processes required for the establishment of arbuscular mycorrhizal symbiosis in land plants are present in streptophyte algae (Delaux *et al.*, 2015). Indeed, zygnematophyceaen orthologs of calcium- and calmodulin-dependent protein kinase (CCaMK; a key component of the symbiosis toolkit) rescued *Medicago* mutants defective in this gene and hence mycorrhization (Delaux *et al.*, 2015). Yet, the genetic framework for the downstream signaling has only emerged after diversification of the respective gene families in the ancestor of land plants (Delaux *et al.*, 2015). Thus, building on a conserved chain of upstream signaling, elaborate developmental processes could emerge.

Symbionts are recognized by plants through receptor-like kinases (RLKs), a major family among which are the LysMs (reviewed by Oldroyd, 2013). Pivotal to the establishment of symbioses is the recognition of lipochitooligosaccharides secreted by arbuscular mycorrhizae via specific LysMs (Oldroyd, 2013; Sun *et al.*, 2015). However, LysMs and other RLKs do not only play a role in formation of arbuscular mycorrhizae but are general players during symbiotic and pathogenic interactions. Fabacaean LysM receptors are involved in the recognition of lipochitooligosaccharides of rhizobia to establish colonization and nodulation of their hosts’ roots (Limpens *et al.*, 2003; Madsen *et al.*, 2003; Radutoiu *et al.*, 2003; Arrighi *et al.*, 2006; Smit *et al.*, 2007). In addition to their role in symbiotic interactions, different RLKs, including LysMs, are also the gatekeepers of pathogen responses in angiosperms, recognizing conserved molecular patterns of microbes, such as flagellin or chitin (Gómez-Gómez and Boller, 2000; Kaku *et al.*, 2006; Miya *et al.*, 2007). While we have little insight into RLK function in pathogen recognition outside of angiosperms, important insights have come from the moss *Physcomitrella patens. Physcomitrella patens* is able to sense and respond to chitin and encodes at least one functional chitin sensing CERK1 homolog (Bressendorff *et al.*, 2016). CERK1 is thus one of the few LysM-RLKs that was probably involved in plant immune signaling (upon being challenged by fungi) in the last common ancestor of all land plants.

The genome of *Chara braunii* provided some insight into the evolutionary history of LysM receptors (Nishiyama *et al.*, 2018). It appears that the common ancestor of *C. braunii* and land plants may have had a single LysM member and that each lineage has undergone their specific family expansions. Without functional studies, it is thus equally plausible that LysMs of streptophyte algae function in response to pathogens or symbionts—or both; hence, the same ambiguity currently applies to inferences of LysM function in the earliest land plants. What we know, however, is that streptophyte algae have the genetic toolkit to sense microbial associates, mutualistic or pathogenic microorganisms. Streptophyte algae associate with an entire microbiome of fungi and bacteria (Knack *et al.*, 2015) and we can predict, based on the presence of LysMs, that they are able to recognize and respond to these microbes in some manner. A similar prediction may be made for the earliest land plants.

It was argued that fungi evolved the ability to degrade plant material before land plants came to be (Berbee *et al.*, 2017). This suggests, as one would expect, that not all of the earliest land plants’ microbiome was friendly; they might have been assaulted by foes that their ancestor already faced in freshwater habitats. Another type of pathogen receptor is shared by streptophyte algae and land plants: the intracellular resistance (*R*) genes that encode proteins with nucleotide-binding domain and leucine-rich repeat (NBS-LRR) domains (Gao *et al.*, 2018; Han, 2019). Yet, again, streptophyte algae seem to have evolved different domain associations from land plants and only a few land plant-like proteins of the toll interleukin 1 receptor (TIR)-NBS-LRR class are present in some streptophyte algae (Gao *et al.*, 2018; Han, 2019).

Only through detailed studies of the interactions between streptophyte algae and their microbiome (see Delaux *et al.*, 2015; Knack *et al.*, 2015; Gao *et al.*, 2018; reviewed in de Vries *et al.*, 2018b; Han, 2019) will we be able to infer the plant–microbe interaction toolkit of the earliest land plants. Yet as to the exact components that the earliest land plants used, the power of inference based on extant systems might be limited: receptor families have undergone many lineage-specific expansions and reductions, and are co-opted for their specific environment, which—most importantly—is co-evolving with their hosts. In contrast, the evolution of the responses to abiotic stressors may be reconstructed more easily, as the abiotic environment—albeit dynamically changing—is not shaped by an evolutionary arms race with other organisms. This touches upon a series of traits that must have been present in the earliest land plants and enabled them to overcome abiotic terrestrial stressors.

### Streptophytic stress signaling: the evolution of an essential prerequisite for the conquest of land

Dwelling on *terra firma* comes with various challenges. Foremost among these are various abiotic stressors, including drought and desiccation, UV irradiation, and rapid changes in temperature—but also changes in substrate quality such as pH, salinity, and nutrient variation. Land plants have evolved an elaborate stress response framework. This framework includes the perception of stressors, signal transduction involving ubiquitous molecules such as reactive oxygen species (ROS) and specific phytohormones such as ABA, and finally the appropriate adjustment of the physiology of the plant cell—all of which further hinges on the duration of a stress (Kranner *et al.*, 2010). The connection between stress input and adjustment is mediated by the plant perceptron. The plant perceptron is a layered network of input signals converging in signal transduction pathways that target regulators of plant growth and physiology (Scheres and van der Putten, 2017). This response system can be considered as an additional defining trait of embryophytes. It is conceivable that the roots of this trait run deeper—already the earliest land plants had to successfully overcome the challenges that the terrestrial stressors posed in order to first colonize land and then radiate on it.

Streptophyte algae are now known to have genes for stress response that were previously thought to be characteristic for land plants. While the functions of many of these genes have not yet been tested, their mere presence warrants attention. These genes represent the ancestral gene pool from which the embryophytic genes with functions in stress response have evolved. When exactly the embryophytic function (if there is a unique function among all embryophytes) evolved can only be identified by a combination of comparative functional and bioinformatic approaches across and outside the monophyletic land plants. Independent of the function they hold nowadays in streptophyte algae, it is these genes that are prime candidates for being part of the ‘terrestrialization toolkit’. The idea is that this toolkit entailed adaptive and exaptive genes that provided a selective advantage during plant terrestrialization (see also de Vries and Archibald, 2018). Standing out among these candidates is the notion that streptophyte algae have the genetic capacity to utilize phytohormone-based signaling pathways (Delaux *et al.*, 2012; Hori *et al.*, 2014; Ju *et al.*, 2015; Van de Poel *et al.*, 2016; Ohtaka *et al.*, 2017; de Vries *et al.*, 2018a; Mutte *et al.*, 2018; Nishiyama *et al.*, 2018). In land plants, phytohormones interact in converging networks of regulatory circuits (Kohli *et al.*, 2013) many of which are part of the plant perceptron that is put to use when dealing with environmental cues (Scheres and van der Putten, 2017).

Auxin is arguably the most famous phytohormone. Polar transport of auxin is a cornerstone in the development of land plants (e.g. Friml *et al.*, 2003) and it orchestrates developmental processes throughout the plant body (Weijers and Wagner, 2016). In recent years, auxin has further been recognized to be a major player in the adjustment of the cell biology of plants to stress cues (Naser and Shani, 2016; Blakeslee *et al.*, 2019). Auxin has been detected in streptophyte algae and its polar transport has been described in Klebsormidiophyceae and Charophyceae—suggesting that polar auxin transport was an early invention (Cooke *et al.*, 2002; Boot *et al.*, 2012; Hori *et al.*, 2014; Ohtaka *et al.*, 2017). It appears however, that polar auxin transport is not present in all streptophyte algal lineages. Indeed, the *Kf*PIN homolog of *Klebsormidium flaccidum* is an auxin-specific transporter, which in heterologous experiments localizes in a non-polar manner in the plasma membrane; in *K. flaccidum*, *Kf*PIN is localized at the peripheral plasma membrane rather than at positions of cell–cell contact (Skokan *et al.*, 2019). In contrast, in *Chara vulgaris*, auxin transporters appear to be localized in a polar manner (Żabka *et al.*, 2016), which Skokan *et al.* (2019) hypothesized to have evolved convergently in the algae and embryophytes. Further, the canonical auxin perception and transduction pathway probably first emerged in land plants, as inferred from transcriptional responses and the presence of the required signaling components (Mutte *et al.*, 2018).

Streptophyte algae are predicted to have pathways for utilizing phytohormones that are predominantly known as relevant for the response to environmental cues. Foremost among those is the homologous genetic framework for the signaling cascade that all land plants use in ABA-mediated responses (Umezawa *et al.*, 2010; Eklund *et al.*, 2018). ABA is known as a major stress phytohormone; the signaling ABA triggers is involved in responses to abiotic cues such as salt, drought, and temperature (reviewed by Ingram and Bartels, 1996; Shinozaki *et al.*, 2003; Yoshida *et al.*, 2014; Fahad *et al.*, 2015). This ABA signaling cascade consists of a three-component core signaling module (see Cutler *et al.*, 2010) that is a chain of negative regulation: when ABA is present, it binds to a receptor of the PYRABACTIN RESISTANCE1/PYR1-LIKE/REGULATORY COMPONENTS OF ABA RECEPTOR (PYR/PYL/RCAR) family that inhibits the PROTEIN PHOSPHATASE 2C (PP2C) proteins that usually would prevent activity of the SUCROSE NONFERMENTING 1-RELATED PROTEIN KINASEs (SnRKs). The SnRKs are the components that activate the downstream targets, such as ion channels or TFs (Furihata *et al.*, 2006; Geiger *et al.*, 2009). The presence and functional conservation of the interaction of PP2Cs, SnRKs, and downstream targets such as ion channels have been investigated through experimental work for proteins of the streptophyte alga *Klebsormidium* (Holzinger and Becker, 2015; Lind *et al.*, 2015; Shinozawa *et al.*, 2019).

Recently, a transcript probably coding for an orthologous protein of the land plant PYL was detected in *Zygnema circumcarinatum* (de Vries *et al.*, 2018a). The presence of PYR/PYL/RCAR homologs in Zygnematophyceae was recently corroborated by the publication of the first two genomes of Zygnematophyceae (Cheng *et al.*, 2019). Cheng *et al.* (2019) found PYR/PYL/RCAR homologs in one (*Mesotaenium endlicherianum*) of the two zygnematophyceaen genomes. Interestingly, in the second zygnematophyceaen genome the authors analyzed, that of the newly described alga *Spirogloea muscicola* (Cheng *et al.*, 2019), no PYR/PYL/RCAR homolog was found; the same applied to an independent genome study of another Zygnematophyceae, *Penium margaritaceum* (Jiao *et al.*, 2019, Preprint). Hence, the genes of the PYR/PYL/RCAR family were probably gained at the base of the monophyletic group of Zygnematophyceae and land plants (see also the excellent discussion in Cuming, 2019). Functional studies of the protein encoded by the homologous PYL gene found in *Z. circumcarinatum* have shown that *Zc*PYL does interact with the downstream PP2Cs—but that it does so in an ABA-independent manner (Sun *et al.*, 2019). The exact regulatory function of PYL:PP2C:SnRK is thus an open question (see also discussions in the last section of this manuscript). It is, however, clear that the presence of these genes has offered fertile ground for the evolution of the canonical ABA signaling cascade that we know from land plants.

### Carrying out the essential work downstream of the signal transduction cascades: streptophytic stress responses

Chemodiversity in secondary metabolites is a key trait of land plants. It has been speculated that such a breadth of secondary metabolites has been critical for the success of embryophytes in the challenging environment of *terra firma* (Weng, 2014). A prime source from which a plethora of embryophytic secondary metabolites emerges is the phenylpropanoid pathway, which is the backbone for a range of compounds that can be associated with any abiotic (terrestrial) stressor imaginable (Dixon and Paiva, 1995; Vogt, 2010). All land plants—bryophytes and tracheophytes—use the phenylpropanoid pathway when challenged with stressors (Wolf *et al.*, 2010; Oliva *et al.*, 2015; Albert *et al.*, 2018; Clayton *et al.*, 2018; Carella *et al.*, 2019). Indeed, the phenylpropanoid pathway was thought to have emerged at the base of the clade of embryophytes, thus being an early adaption of embryophytes (Emiliani *et al.*, 2009; Weng, 2014). However, a homologous genetic framework for many components of the core phenylpropanoid pathway was found in many streptophyte algal species when several transcriptomic data sets and one genome were cumulatively investigated (de Vries *et al.*, 2017). This suggests that streptophyte algae could in general be able to synthesize phenylpropanoids—and possibly downstream derivatives, which have been previously reported as a land plant invention (de Vries *et al.*, 2017). Indeed, biochemical and histochemical data accumulated over the years indicate that lignin-like components are present in the cell walls of several streptophyte algae—most prominently in the genus *Coleochaete* (Delwiche *et al.*, 1989; Sørensen *et al.*, 2011). In land plants, the production of lignins can be considered a route that is distal to—but hinging on—the core part of the phenylpropanoid pathway (Vanholme *et al.*, 2012). Together with the genes found in many streptophyte algae, this suggests that the capacity to use core and peripheral routes of the phenylpropanoid pathway arose before phragmoplastophytes emerged. Interestingly, Jiao *et al.* (2019, Preprint) detected flavonoids in the Zygnematophyceae *Penium margaritaceum* despite the absence of homologous genes that might code for the two required upstream enzymes of the core phenylpropanoid pathway in its genome. Altogether, it is likely that the phenylpropanoid pathway was already present—in some fashion—in the earliest land plants. There, it might have acted in the production of compounds that warded off terrestrial stressors. Further, this provided fertile genetic ground for likely gene duplication-based diversification of the pathway routes and the chemodiversity it yields (see Niklas *et al.*, 2017 for an erudite explanation).

High irradiances are a prime challenge in the terrestrial environments. What do extant streptophyte algae tell us about how the earliest land plants might have overcome these? UV irradiances could have been shielded by phenolic compounds produced by the phenylpropanoid pathway (Popper *et al.*, 2011; de Vries *et al.*, 2017), including the recently detected flavonoids (Jiao *et al.*, 2019, Preprint). It is hence conceivable that phenylpropanoid-derived metabolites were part of the UV screen of the earliest land plants.

The green lineage appears to have a nuclear-encoded plastid proteome that is particularly moldable by stressors (Knopp *et al.*, 2019, Preprint). In land plants, a tight communication between the plastid and nucleus adjusts the plastid proteome under photophysiologically challenging irradiances via operational retrograde signaling (see also Chan *et al.*, 2016). The evolution of such a tight framework of communication was probably a major benefactor of plant terrestrialization (de Vries *et al.*, 2016). In fact, genes for a land plant-like retrograde signaling—including GENOMES UNCOUPLED 1 (GUN1) and SAL1—from the plastid to the nucleus seem to have emerged at the base of phragmoplastophytes (de Vries *et al.*, 2018a; Nishiyama *et al.*, 2018; Zhao *et al.*, 2019); the earliest land plants probably used these genes in an operational retrograde signaling network for attuning their plastid to terrestrial stressors.

A well-conserved mechanism for protection against high light is the mitigation of excess energy that is generated during photosynthesis through non-photochemical quenching (NPQ). This mechanism involves the dissipation of excess excitation energy of excited singlet chlorophyll as heat, thus preventing ROS formation and reducing the risk of photooxidative damage—especially to the damage-prone PSII (Aro *et al.*, 1993; Müller *et al.*, 2001). NPQ mechanisms exhibit great variability in green algae (Goss and Lepetit, 2015; Christa *et al.*, 2017). Two proteins, namely LHCSR (light-harvesting complex stress-related protein) and PSBS (photosystem II subunit S), play a major role in NPQ. Both proteins help to activate the fastest NPQ component, qE (energy-dependent quenching), by acting as a pH sensor and inducing conformational changes in LHCII complexes (PSBS, Horton *et al.*, 2000; Li et al., 2000, 2004; LHCSR, Peers *et al.*, 2009; Bonente *et al.*, 2011). For some time, it was thought that in green algae qE depended on the action of the LHCSR proteins (Peers *et al.*, 2009), whereas land plants used another protein of the LHC superfamily, PSBS (Li *et al.*, 2000). Yet, this view was changed by two findings: (i) the chlorophyte *Chlamydomonas reinhardtii* PSBS protein was found to be high light induced—an induction that correlates with NPQ (Correa-Galvis *et al.*, 2016); and (ii) mosses also have an NPQ that depends on the action of LHCSRs and PSBS (Alboresi *et al.*, 2010; Gerotto *et al.*, 2012). This means that the differences in PSBS detection along the trajectory of streptophyte algal evolution (see Gerotto and Morosinotto, 2013) must be seen in a new light—possibly reflecting the notorious difficulties in detecting algal PSBS. Nonetheless, this means that the conclusion of Alboresi *et al.* (2010) holds: the earliest land plants probably had a photoprotection mechanisms that included the action of both LHCSRs and PSBS.

Streptophyte algae that live in terrestrial habitats have remarkable photoprotection capacities. For example, terrestrial *Klebsormidium* can tolerate astoundingly high intensities of light without suffering photoinhibition (Karsten *et al.*, 2016; Pierangelini *et al.*, 2017). Yet, these ecophysiological traits differ when considering multiple representatives of Klebsormidiophyceae (Herburger *et al.*, 2016). Hence, it is likely that such photoprotection mechanisms are particularly moldable by environmental factors, hampering inferences over long evolutionary timescales.

Next to high light, desiccation is a major stressor for streptophyte algae that live in terrestrial habitats (Pierangelini *et al.*, 2019). Indeed, tolerance to both often goes hand in hand. Upon water deficiency, carbon fixation is limited but electron flow continues, leading to possible triplet chlorophyll (^3^Chl*) formation which in turn contributes to the formation of the ROS singlet oxygen (^1^O_2_*) (Frankel, 1984; Müller *et al.*, 2001). Also, under limited carbon fixation, the biosynthesis of photoprotective molecules is impaired, leading to even more ROS formation (Takahashi and Murata, 2008). Terrestrial streptophyte algae such as *Klebsormidium* and *Zygnema* have a remarkable desiccation tolerance (e.g. Pierangelini *et al.*, 2017; Rippin *et al.*, 2017; Herburger *et al.*, 2019). Their tolerance is based on a range of mechanisms such as protective substance production, cell wall remodeling, formation of specialized cells, and many more (for an excellent overview, see Holzinger and Pichrtová, 2016; for more on cell walls and plant terrestrialization, see also Harholt *et al.*, 2016). Even streptophyte algae that are not considered terrestrial, such as *Coleochaete*, can tolerate desiccation when challenged with it in the lab (Graham *et al.*, 2012). Finding the ability to overcome terrestrial stressors in extant streptophyte algae has important implications: it is likely that streptophyte algae have equally found their way onto land multiple times independently (see also Figs 1, 2). Among terrestrial streptophyte algae, there is a mix of completely independent as well as convergent solutions (many of which we illustrate in the next section) to dealing with terrestrial stressors. An example for divergent solutions is that terrestrial *Klebsormidium* deposits callose under desiccation stress while *Zygnema* does not (Herburger and Holzinger, 2015). However, these differences and independent solutions also highlight that streptophyte algae have a moldable genetic framewok for molecular stress responses. Molding this framework eventually formed the network that was also put to use by the earliest land plants.

During the course of evolution, various photosynthetic eukaryotes have found their way onto land. This includes various members of the green lineage that made the wet to dry transition multiple times independently (Lewis and McCourt, 2004). Yet, for example, some diatoms and even cyanobacteria are also terrestrial: photosynthetic organisms that are as distantly related to land plants as can be. These convergently terrestrial organisms must also have found solutions to the challenges of UV, desiccation, high light, etc. In the following we will outline a few of their strategies.

### Is the physiology of streptophytes unique?

Photosynthetic organisms from various lineages have settled on land. Only one became the ancestor of the land plants. In an evolutionary context, it is assumed that there were photosynthesizing eukaryotes and prokaryotes on land long before the origin of embryophytes (Raven and Edwards, 2014). How did they overcome the stressors discussed above in the context of plant terrestrialization? Here, again, the diversity of extant photosynthesizing life on land holds illuminating clues.

Desiccation stress responses in green algae can be divided into different categories depending on their function within the organism (Holzinger and Karsten, 2013). One way of responding to desiccation stress is to avoid it. A prime example for desiccation stress avoidance in extreme environmental conditions are desert biological soil crusts (BSCs). Formation of BSCs is a phenomenon that is well known from chlorophytic and streptophytic green algae (e.g. Belnap and Lange, 2001; Flechtner, 2007; Holzinger and Karsten,2013). Cyanobacteria form BSCs, too. These BSCs are a mixture of sand particles and polysaccharides which are excreted by the cyanobacteria and are able to withstand extreme environmental desiccation, temperature changes, and very high irradiance (Mager and Thomas, 2011; Ferrenberg *et al.*, 2015). Particularly noteworthy is the cyanobacterium *Leptolyngbya ohadii* that is native to deserts (Raanan *et al.*, 2016; Oren *et al.*, 2019). *Leptolyngbya ohadii* employs a range of physiological mechanisms to go through cycles of dehydration and recovery during rewetting: before dehydration, phytochromes and cryptochromes act as signal transmitters, sensing dawn illumination and thus preparing the cell for impending loss of water (Oren *et al.*, 2017). During dehydration, trehalose-based osmotic adjustment and anhydrobiotic protection appear to be key (Murik *et al.*, 2017)—although recent data suggest that their role might be limited (Oren *et al.*, 2019). The dehydration/rewetting cycles induce pronounced transcriptional reprogramming. Genes of *L. ohadii* involved in photoprotection—such as OCP (Leverenz *et al.*, 2015)—were up-regulated during dehydration while genes involved in biosynthesis of photosynthetic components were down-regulated (Oren *et al.*, 2019). Cellular activity (photosynthesis) in *L. ohadii* and other cyanobacteria was measured a short time after rewetting: the results suggested a complex gene regulatory network that is highly adapted to dehydration/rewetting cycles being reflected in swift modulation of the photosynthetic machinery (Bar-Eyal *et al.*, 2015; Oren *et al.*, 2019). It was proposed that intrinsically disordered proteins (IDPs) play a possible role in protecting and stabilizing RNA during desiccation (Oren *et al.*, 2019). IDPs have been studied regarding their role in desiccation tolerance of tardigrades (*Milnesium tardigradum*; Boothby *et al.*, 2017) and might have a similar role in Chloroplastida, too (Sun *et al.*, 2013; Y. Zhang *et al.*, 2018; see also Niklas *et al.*, 2018). Hence, even in terrestrial cyanobacteria—which reside in another domain of life—mechanisms that function similarly in terrestrial chlorophytes and streptophytes can be found.

Protective responses to desiccation stress include formation of a modified cell wall. Some Trebouxiophyceae do so palpably. *Prasiola crispa* subsp*. antarctica* grows, as the name implies, in the supralittoral of Antarctica and possesses a very thick cell wall that protects the alga from osmotic stress during desiccation (Jacob *et al.*, 1992). The cell wall in *Prasiola* contains a large amount of pectin (Jacob *et al.*, 1992). Pectins are important components of the cell wall of land plants and streptophyte algae (Ridley *et al.*, 2001; Sørensen *et al.*, 2011) but have also been shown to be involved in desiccation stress responses in the ulvophyte *Ulva compressa* by providing a flexible cell wall (Holzinger *et al.*, 2015). Under hypoosmotic conditions, the flexible nature of the cell wall prevents the algal cells from swelling excessively, while under hyperosmotic conditions it prevents plasmolysis—a feature that is beneficial not only under desiccation stress but also under salinity stress which is a frequent stressor considering the habitat of *P. crispa* subsp*. antarctica* (Jacob *et al.*, 1992). Hence, both chlorophytes and streptophytes have evolved mechanisms for cell wall modification under desiccation stress.

The ability of algae to form symbiotic interactions is assumed to have been key in the conquest of land by plants (Delaux *et al.*, 2013; Field *et al.*, 2015). While land plants have unique beneficial symbionts (foremost the arbuscular mycorrhiza), other green organisms are not without friends. For example, many species of Trebouxiophyceae are lichen-forming. Lichens are highly resistant to desiccation and are thus able to stay in a dehydrated state for an extended amount of time until rehydration. In order for a lichen to survive under such extreme conditions, the underlying molecular regulatory network must be extremely fine-tuned and adapted to dehydration/rehydration cycles. For example, dehydration of DNA resulting in strand breaks would mean death for the lichen (Dose *et al.*, 1992). Carniel *et al.* (2016) provide a good example for transcriptomic regulation in the lichen photobiont *Trebouxia gelatinosa*; their data reveal a different gene regulatory set-up in various categories such as cell wall modifications, photosynthetic apparatus, or oxidative stress response during dehydration/rehydration cycles (Carniel *et al.*, 2016). In this context, photoprotective processes are crucial because in desiccated photosynthetic tissues there is a high risk of excessive ROS formation (Smirnoff, 1993; Scheibe and Beck, 2011). Rapid adjustments of the antioxidant homeostasis appear to be a mechanisms that lichens employ under desiccation stress (Calatayud *et al.*, 1997; Zorn *et al.*, 2001; Kranner *et al.*, 2003); such antioxidants include zeaxanthin, which can deactivate excited chlorophylls (Jahns and Holzwarth, 2012)—thus substances that are found conserved across plastid-bearing eukaryotes (Dautermann and Lohr, 2017).

The aforementioned PSBS and LHCSR are LHC-like proteins that are key for the response to high light. An additional important group of LHC-like proteins are the early light-induced proteins (ELIPs), which are swiftly up-regulated in response to high light stress as well as also being up-regulated in response to dehydration stress and by ABA (Bartels *et al.*, 1992; Pötter and Kloppstech, 1993; Zeng *et al.*, 2002; Dinakar and Bartels, 2013). ELIPs are hallmark stress-responsive proteins in a variety of oxygenic photosynthetic organisms from cyanobacteria, to algae, to land plants (Heddad and Adamska, 2002). Hutin *et al.* (2003) demonstrated that ELIPs have an essential photoprotective role by using the *A. thaliana* mutant *chaos*, which is not able to translocate LHC-type proteins such as ELIP via the chloroplast signal recognition particle (CpSRP) pathway to the thylakoid membranes (see also Hutin *et al.*, 2002). The authors observed leaf bleaching and photooxidative damage in *chaos* mutants challenged with high-light cues. The data of Hutin *et al.* (2003) thus revealed the photoprotective role of ELIPs. It was further suggested that ELIPs have an influence on chlorophyll biosynthesis, thus indirectly preventing chlorophyll accumulation under high light conditions and thereby also preventing ROS formation (Hutin *et al.*, 2003; Tzvetkova-Chevolleau *et al.*, 2007).

The formation of sunscreens is a powerful protection against UV irradiance. Above, we illustrated how the biosynthesis of phenylpropanoid-derived metabolites is involved in this mechanism in streptophytes—and discussed how such metabolites might have been critical for streptophyte terrestrialization. Yet, other terrestrial organisms have sunscreens, too. In some cyanobacteria, scytonemin acts as a very potent UVA sunscreen that protects the cells from near UV and blue radiation (Gao and Garcia-Pichel, 2011). It accumulates as a stable pigment in extracellular polysaccharide sheaths of cyanobacteria and possesses various convenient characteristics. Scytonemin changes dependent on the redox status, stays active even under physiological inactive conditions, and is capable in performing strong absorption in the UVA range due to its ring structure with conjugated double bonds (Garcia-Pichel and Castenholz, 1991; Proteau *et al.*, 1993; Gao and Garcia-Pichel, 2011). The pigment is typically found in biological soil crusts or epilithic biofilms (Gao and Garcia-Pichel, 2011). While many questions regarding its biosynthesis and regulation remain, the exposure of cyanobacterial cells to UVA irradiance, however, appears to be correlated with scytonemin production (Garcia-Pichel and Castenholz, 1991; see also Ehling-Schulz *et al.*, 1997).

In chlorophyte algae, mycosporine-like amino acids (MAAs) act as sunscreens when exposed to solar UV radiation. For example, an accumulation of MAAs was found to be advantageous against UV irradiance in the aeroterrestrial green algae *Stichococcus* sp. and *Chlorella luteoviridis* when compared with two green algae from soil with a different MAA set-up (Karsten *et al.*, 2007). Such aeroterrestrial species possess a unique type of MAA (324 nm MAA) which is only found in Trebouxiophyceae—including the alga *P. crispa* spp. *antarctica* (Hoyer *et al.*, 2001; Karsten *et al.*, 2005). In the streptophyte algal class of Klebsormidiophyceae, we also find the production of 324 nm MAA—yet, despite their similar absorption spectrum, these are different substances (Kitzing and Karsten, 2015). This provides yet another intriguing example of convergent evolution.

So, what makes streptophytes special? There are multiple lineages of algae that made their way onto land by mastering its stressors. Post-hoc, we see that only the progenitors of the last common ancestor of embryophytes gave rise to a lineage that globally conquered land. Why do only they dominate the terrestrial flora? A specific (embryophyte-like) physiology that aided them in dealing with stressors will not be the only reason. However, it has to be part of the answer. Dealing with terrestrial stressors was not only under selection in the earliest land plants but is under selection still. Like the core processes in energy metabolism (such as the citrate cycle) that are essential, core response mechanisms to stressors are continually tested by adverse terrestrial conditions and are essential for survival in the terrestrial habitat. The question is which building blocks of that core were present in the earliest land plants.

### Rewiring of an ancient molecular physiology was a likely facilitator of the success of land plants

The responses of land plants to stressors hinges on complex regulatory networks. In such stress-relevant networks, nodes are genes/proteins that are connected by edges that circumscribe an interaction—such as phosphorylation and transcriptional activation—that are triggered and/or modulated by environmental stimuli. Dissecting ancient pathways and setting them into context with land plant data enables us to trace the evolution of such networks across the streptophyte tree of life. Correlative data, such as genomes, have indicated the presence of many genes (the nodes) that could act in stress-relevant regulatory networks—but what about the wires that connect them (the edges)? To understand these, more involved functional analyses are required.

ABA is a key modulator of stress response. Above, we outlined that streptophyte algae have most—and Zygnematophyceae all—homologous genes of the canonical cascade that land plants use for ABA signaling (de Vries *et al.*, 2018a; Cheng *et al.*, 2019; Sun *et al.*, 2019). What does this mean for the evolution of the laying of the wires that underpin ABA signaling? Although *Klebsormidium* completely lacks a gene for the ABA receptor, Holzinger *et al.* (2014) found the other components (PP2C, SnRK2s, and AREBs) to be responsive to drought (see also Holzinger and Becker, 2015). Furthermore, the genes of *Klebsormidium* that are homologous to ABA signaling components complement the respective mutants of *A. thaliana* and *P. patens* in heterologous experiments (Lind *et al.*, 2015; Shinozawa *et al.*, 2019); for example, expressing *Klebsormidum nitens* SnRK2.6 in *A. thaliana* protoplasts that are deficient in SnRK2.2, 2.3, and 2.6 (*snrk2.2/2.3/2.6* triple mutants) rescues the transduction of ABA-induced gene expression (Lind *et al.*, 2015). Hence, the specific wires of the interaction required for the ABA signaling cascade that are downstream of the ABA–PYL interaction appear to be conserved between Klebsormidiophyceae and land plants. This means that the cascade is probably hundreds of millions of years older than its bona fide ABA dependency mediated by PYLs. Recently, another piece was added to the puzzle of the evolution of the ABA signaling cascade: through *in vitro* and heterologous work, Sun *et al.* (2019) showed that the PYL homolog found in *Z. circumcarinatum* does regulate its downstream target—the PP2Cs. Yet, their data also show that this regulation happens in an ABA-independent manner. What this means is that the bona fide ABA signaling cascade, which consists of PYLs:PP2Cs:SnRKs, has evolved in a modular fashion—with components being successively plugged in. The ABA dependency of the cascade—another addition—has probably evolved along the trajectory from the earliest land plants to the last common ancestor of land plants (see also the discussion on this topic in Sun *et al.*, 2019). Whether there is nonetheless an ABA dependency of parts of the signaling cascade in algae mediated through other means—such as the *ABA NON-RESPONSIVE* kinases that are only present in non-flowering plants (Stevenson *et al.*, 2016)—remains to be investigated. However, it offers an explanation for how conserved stress tolerance-conferring mechanisms such as LEA accumulation can be part of the same cascade that we now think of as ABA mediated: they might have been part of the downstream response triggered by the same regulatory cascade conserved for hundreds of millions of years, in which they have been functional and riding along since before the signal transduction chain was under the control of ABA.

The signaling network that mediates the action of the growth phytohormone auxin has a similar story to tell. A series of recent studies [Flores-Sandoval *et al.* (2018); Mutte *et al.* (2018); Martin-Arevalillo *et al.* (2019)] proposed an evolutionary history that features a modular build-up for the nuclear auxin response pathway that is mediated by AUXIN RESPONSE FACTORS (ARFs), the TRANSPORT INHIBITOR RESPONSE 1/AUXIN SIGNALLING F-BOX (TIR/AFB), AUXIN/INDOLE-3-ACETIC ACID PROTEIN (AUX/IAA) system: class C ARFs are present in streptophyte algae—and perhaps were even present in the last common ancestor of streptophytes (see also Wang *et al.*, 2019); the common ancestor of Coleochaetophyceae, Zygnematophyceae, and land plants gained the single co-ortholog (A/B ARF) of the classes of A and B ARFs known from land plants. In the absence of TIR/AFB and AUX/IAA, these algal ARFs act in an auxin-independent manner (Martin-Arevalillo *et al.*, 2019; see also Mutte *et al.*, 2018). In land plants, the A/B ARF diverged into the class A and class B ARFs (Flores-Sandoval *et al.*, 2018; Mutte *et al.*, 2018; Martin-Arevalillo *et al.*, 2019). Onto this diversified system, the TIR/AFB and AUX/IAA were plugged, rendering the whole system auxin dependent (Flores-Sandoval *et al.*, 2018; Mutte *et al.*, 2018; Martin-Arevalillo *et al.*, 2019). Thus, adding one regulatory mechanism upstream was sufficient to turn a conserved gene regulatory cascade into a phytohormone-dependent cascade. Similar to the aforementioned case of ABA-mediated signaling, gaining such phytohormone dependency probably occurred along the trajectory between the earliest land plants and the last common ancestor of land plants.

Recent functional studies on the liverwort model system *M. polymorpha* have illustrated how a different type of modular evolution of a phytohormone pathway can occur—in this case not concerning a protein that is plugged in, but a different effector molecule. Of course, such changes ultimately hinge on differences on the protein level, too; that is, in the binding pocket for the input molecule. Monte *et al.* (2018) investigated the origin of jasmonic acid (JA) perception by focusing on the COI1 receptor. Instead of sensing JA-Ile, the COI1 protein of *M. polymorpha* senses dinor-12-oxo-phytodienoic acid (dinor-OPDA), which emerges from an earlier branching point in the JA biosynthesis pathway (Monte *et al.*, 2018). Hence, while the entire pathway for the canonical, JA-Ile-based, perception of JA is present in the bryophyte *M. polymorpha*, it does not work in the same way as we know it from angiosperms. Plugging in a different molecule at the uppermost layer of the signaling cascade hence has the ability to change the entire co-evolutionary relationship between phytohormone biosynthesis and its perception. Even more extreme cases of rewiring can be expected to have taken place in streptophyte algae, which are hundreds of millions of years divergent from land plants. Thus, the gain of a specific phytohormone dependency can also entail shifts in input signal. Illuminating whether such shifts in input signal underpinned the evolution of other phytohormone signaling cascades (such as ABA) is an exciting avenue for future research.

Rewiring is not limited to signaling pathways. Secondary metabolites of land plants are known for their chemodiverse and lineage-specific—sometimes species-specific—secondary metabolic fingerprints (see, for example, Dudareva *et al.*, 2004). During the course of evolution, the same building block-producing backbone pathways have been rewired to give rise to tens of thousands of different secondary metabolites. For example, Berland *et al.* (2019) recently showed that the liverwort model system *M. polymorpha* produces a novel class of anthocyanins, which they termed auronidins. Auronidins appear to be derived from anthocyanin but emerge from a novel route via aurones (Berland *et al.*, 2019). This highlights the complexities one has to tackle when inferring the secondary metabolism of the earliest land plants. Since the genes for the phenylpropanoid biosynthesis pathway are present in streptophyte algae (de Vries *et al.*, 2017) and building blocks have been detected even in chlorophytes (Goiris *et al.*, 2014), we can expect that the earliest land plants used a plethora of secondary metabolites derived from these routes. Inferring the routing of the pathways, however, requires more than the mere knowledge of the presence of some genes or some metabolites. A functional dissection of the biosynthetic pathways across the streptophyte tree of life—especially in the streptophyte algae—is needed.

### Conclusion

The lineage of embryophytes has conquered land. Only through a fortuitous combination of traits did they succeed in this conquest. These traits include complex networks for stress response that are, in their elaboration, probably limited to land plants. Yet, often, the integral components and/or decisive nodes and wires of these networks were already present—they represent complete and functioning building blocks ready for co-option. Moreover, as of yet, we do not know how most of these components function in their own environment; that is, how are they wired in streptophyte algae? It may very well be that the present components have the same functions as in land plants, yet their interactors may be different in streptophyte algae (e.g. different receptor specificity or different downstream components). Likewise, a completely different function for the streptophyte algal components underlying a network is also entirely possible. One thing, however, is very clear; these conserved proteins that participate in the stress signaling networks in land plants will not be without a function in streptophyte algae. Comparative studies that dissect the routing of upstream (e.g. signaling) and downstream (e.g. biosynthesis of stress protectants) stress response pathways across streptophytes will illuminate how their co-options were realized in the earliest land plants.
